# The Open-Door System

**Published:** 1888-04-07

**Authors:** 


### The Open-Door System.

As we anticipated in our notice of the Glasgow Asylum, Dr. Yellowlee's opinion of the open door system has not passed unchallenged. We gladly insert the following from a Scotch correspondent, who seems to take a very sensible view of the situation:

"In his report, the Medical Superintendent refers to the vexed question of the increased liberty given to patients in some Scotch asylums by what is known as the 'open-door system.' His remarks on this subject plainly indicate his disapproval of 'unwise indulgence,' and he claims that in his asylum 'the lines of restriction are as wide as welfare permits.' Dr. Yellowlee's long and highly honourable career as an asylum physician entitle his opinions to all respect, and he is far from being the only superintendent even in Scotland who holds such views, but it must be admitted that several superintendents in this country have in recent years considerably relaxed the rules elsewhere hitherto looked on as absolutely essential, and that they have done so with apparent success. These latter, however, have erred in attaching too great importance to their small departures from established usage, and that they have gone an unwarrantable length in assuming, as they frequently do, a national importance for what they have taught our southern neighbours to call the 'Scotch system,' is proved by the remarks above referred to from the pen of the administrative head of one of the most important and successfully-managed institutions for the insane in the country.

"I believe the difference in the amount of liberty accorded is less wide than appears in controversial statements. Some persons have called for interference on the part of the central authorities, and have, with inadequate foundation, accused those authorities of too facile sympathy with new and unsafe methods. In my opinion, the attitude of the General Board of Lunacy in Scotland has been wisely chosen. By allowing some latitude to the responsible heads of asylums in such matters, changes that may prove important advances have a chance of trial. The open-door system may prove a failure. The reports of various asylums where it has been adopted show that, so far, there is something to be said for it. I refrain from expressing an opinion on the controversy; but to assert that the Scotch Commissioners in Lunacy connive at what is proved to be rash and injurious is beyond all reason."

